# Rapid gastrointestinal loss of Clostridial Clusters IV and XIVa in the ICU associates with an expansion of gut pathogens

**DOI:** 10.1371/journal.pone.0200322

**Published:** 2018-08-01

**Authors:** Alexandra E. Livanos, Erik J. Snider, Susan Whittier, David H. Chong, Timothy C. Wang, Julian A. Abrams, Daniel E. Freedberg

**Affiliations:** 1 Division of General Medicine, Columbia University Medical Center, New York, NY, United States of America; 2 Department of Pathology and Cell Biology, Columbia University Medical Center, New York, NY, United States of America; 3 Division of Allergy, Pulmonary, and Critical Care Medicine, Columbia University Medical Center, New York, NY, United States of America; 4 Division of Digestive and Liver Diseases, Columbia University Medical Center, New York, NY, United States of America; University of Illinois at Urbana-Champaign, UNITED STATES

## Abstract

Commensal gastrointestinal bacteria resist the expansion of pathogens and are lost during critical illness, facilitating pathogen colonization and infection. We performed a prospective, ICU-based study to determine risk factors for loss of gut colonization resistance during the initial period of critical illness. Rectal swabs were taken from adult ICU patients within 4 hours of admission and 72 hours later, and analyzed using 16S rRNA gene sequencing and selective culture for vancomycin-resistant *Enterococcus* (VRE). Microbiome data was visualized using principal coordinate analyses (PCoA) and assessed using a linear discriminant analysis algorithm and logistic regression modeling. 93 ICU patients were analyzed. At 72 hours following ICU admission, there was a significant decrease in the proportion of Clostridial Clusters IV/XIVa, taxa that produce short chain fatty acids (SCFAs). At the same time, there was a significant expansion in *Enterococcus*. Decreases in Cluster IV/XIVa Clostridia were associated with loss of gut microbiome colonization resistance (reduced diversity and community stability over time). In multivariable analysis, both decreased Cluster IV/XIVa Clostridia and increased *Enterococcus* after 72 hours were associated with receipt of antibiotics. Cluster IV/XIVa Clostridia, although a small fraction of the overall gastrointestinal microbiome, drove distinct clustering on PCoA. During initial treatment for critical illness, there was a loss of Cluster IV/XIVa Clostridia within the distal gut microbiome which associated with an expansion of VRE and with a loss of gut microbiome colonization resistance. Receipt of broad-spectrum antibiotics was associated with these changes.

## Introduction

The gastrointestinal microbiome plays crucial roles in maintenance of the gut mucosal barrier, immunity, and protection against infection [1]. In patients who are critically ill, the normal gut microbiome substantially shifts, likely impacted both from critical illness itself and from antibiotics and other exposures common to the intensive care unit (ICU) [2].

Loss of colonization resistance, the ability of the normal gut microbiome to resist the expansion of pathogens, is thought to be a risk factor for ICU-acquired infections and may contribute to the organ dysfunction seen during sepsis [3, 4]. Typical hospital pathogens such as Gram-negative bacilli and vancomycin-resistant *Enterococcus* (VRE) are more prevalent in ICU patients compared to healthy individuals [5]. Fecal microbial diversity, a proxy measure for colonization resistance, is low at the time of ICU admission [6] and further declines with treatment; prolonged critical illness and the accompanying antibiotics may lead to a state of ultra-low diversity and near-complete loss of colonization resistance [7]. In bone marrow transplant (BMT) patients, loss of fecal microbial diversity is followed by domination with enteric pathogens including VRE and increased risk for subsequent infection [8, 9].

The specific changes that lead to loss of normal colonization resistance are uncertain. Loss of obligate anaerobes may facilitate the acquisition of pathogens and has been observed as a consequence of antibiotics [10–12]. In particular, loss of Clostridial Clusters IV/XIVa, which produce butyrate and other short chain fatty acids (SCFA), has been correlated with susceptibility to enteric pathogens [13]. In small ICU-based studies, loss of anaerobes has been associated with increases in *Enterococcus* and *Staphylococcus* [14]. Large shifts away from anaerobic commensals have been seen in the ICU and have been associated with worse clinical outcomes [15, 16]. However, it is unknown exactly which bacteria or combination of bacteria are lost, when, and how they may maintain colonization resistance.

We conducted this study to determine the key fecal microbial changes during the period immediately following ICU admission. Our goal was to identify potentially protective bacterial taxa that might be lost during this initial ICU treatment period, and to test which clinical factors were associated with these changes.

## Materials and methods

### Patient population

Adults ≥18 years old were considered for the study if they were newly admitted to any one of 5 distinct medical or surgical ICUs and could be reached within 4 hours of ICU admission. Patients were excluded for prior *Clostridium difficile* infection (within 90 days), bacterial bloodstream infections (within 30 days), or ICU admissions (within 30 days). Patients were also excluded if both study assessments could not be completed due to death, discharge, or patient refusal. Informed consent was obtained from all subjects or from appropriate surrogates when subjects lacked capacity. Default consent was written although verbal (telephone) consent was accepted when subjects lacked capacity and the appropriate surrogate was not available in person. This study was approved by the institutional review board of Columbia University.

### Study design

This was a prospective, ICU-based study consisting of two assessments. The initial study assessment was made within 4 hours of ICU admission and the second and final study assessment was performed 72 hours later (+/- 4 hours).

### Study assessments

At each study assessment, samples were taken and information was gathered. Two duplicate deep rectal flocked nylon swabs [17] (Copan Diagnostics, Murrieta, CA) were collected and the following information was gathered at the bedside: vital signs, use of life support devices (e.g., mechanical ventilation, hemodialysis), and the Glasgow Coma Scale [18]. Demographic information, laboratory data, and data related to interventions performed in the ICU between study visits was extracted from the electronic medical record. For laboratory data, test results were used from the first venous blood draw in the ICU (corresponding to the first study assessment) and from a venous blood draw either at or immediately preceding the 72 hour mark (corresponding to the second study assessment). ICU interventions were recorded including antibiotics (any dose or duration), proton pump inhibitors, mechanical ventilation, hemodialysis (either intermittent or continuous), and enteral feeding (by mouth or by naso-enteric tube). Clinical and laboratory data were used to estimate acute severity of illness (APACHE IV) [19].

### 16S rRNA gene sequencing

The first of each pair of duplicate swabs was frozen at -80°C for batched DNA extraction at the end of the study (PowerFecal, MoBio, Carlsbad, CA). Polymerase chain reaction was performed targeting the V4 hypervariable region of the 16S ribosomal RNA gene with primers derived from the human microbiome project (primers and protocol in Supplementary materials and methods) [20–22]. After PCR of V4 hypervariable region with above primers/protocol, samples were pooled and purified with the QIAquick PCR kit (Qiagen, Valencia, CA) and library quantification performed using a KAPA Library Quantification Kit (Kapa Biosystems, Wilmington, MA). Sequencing of the 16S ribosomal RNA gene V4 region was performed using the Illumina HiSeq 4000 platform (Illumina, San Diego, CA).

Singleton reads were discarded and read pairs were merged, trimmed, and filtered for quality using *mothur* [23]. Subsequently singleton contigs were discarded. Greengenes [24] was used as a reference database with additional sequences of interest retrieved from the National Center for Biotechnology as needed. Clustering of taxonomic units was made at 97% sequence similarity using USEARCH [25] and taxonomic assignments were made using *mothur* [23]. FastTree2 [26] was used to generate a phylogenetic tree of the contigs.

The QIIME pipeline [27] was used to calculate α-diversity metrics (Shannon diversity index and number of observed Operational Taxonomic Units (OTUs)) and β-diversity (unweighted UniFrac distances [28]), and to generate principal coordinate analysis (PCoA) plots based on unweighted UniFrac distances. For α- and β-diversity metrics, samples were rarefied to 50,000 to allow for calculations based on even number of sequences per sample. Microbiome stability was calculated as the within-individual unweighted UniFrac distance from ICU admission to 72 hours later where larger UniFrac distances represented greater sample divergence over time and thus less stability. Sequencing data from the study is available in the short read archive section of the National Center for Biotechnology Information (accession number SRP149563).

### VRE-selective stool culture

At both study assessments, duplicate rectal swabs were performed and were inoculated into soy broth with 20% glycerol media at the bedside. After gentle mixing, these swabs were plated on VRE-selective chromogenic media (Remel, Lenexa KS) and incubated aerobically at 33–37°C. After 24 hours, colonies were inspected for characteristics, morphology, and color. Results were classified categorically according to the manufacturer’s instructions as VRE present (including *E*. *faecalis* or *E*. *faecium*) versus VRE absent.

### Statistical approach

Comparisons of clinical data were performed using chi-squared tests, Fisher’s exact test (when expected cell counts were ≤5), or paired *t* tests for continuous variables.

Analysis of gut microbiome data was performed using a stepwise approach. First, an untargeted hierarchical linear discriminant analysis (LDA) effect size algorithm (LEfSe) [29] was used to identify significant within-individual changes, comparing the time of ICU admission versus 72 hours later. LEfSe tests for non-parametric differences in features (Kruskal-Wallis) and uses these results for paired testing for a difference between pre-specified classes (Wilcoxon rank-sum). A LDA cut-off of ≥ 2.0 was applied and all taxa were specified at the lowest possible hierarchical level. LEfSe can exaggerate the importance of scarce taxa, so this analysis was limited to taxa that had both significantly changed and that had a relative abundance ≥0.005 in any sample. Second, the univariable relationships between clinical data and taxa identified as significantly changed by LEfSe were examined. For this analysis, LEfSe taxa were divided into quartiles based on relative abundance after 72 hours. Third, a multivariable model was constructed for these relationships using ordered logistic regression. Variables considered for this model had an unadjusted relationship with LEfSe taxa at the p<0.10 level. To further explore the data, sensitivity analyses were performed testing for relationships between dynamic clinical changes and altered LEfSe taxa within the final model (e.g., *worsening* APACHE IV).

*Post hoc* clustering analyses were performed selecting cut-offs based on the observed data. To test for differences in α-diversity, the Wilcoxon U test and was used with GraphPad Prism 7 (La Jolla, CA). To test for differences in β-diversity, unweighted UniFrac distances were used with QIIME after a Bonferroni-adjusted Monte Carlo simulation [27]. STATA 14 (StataCorp, College Station, TX) was used for multivariable modeling and other significance testing. All tests were performed 2-sided at the alpha 0.05 level.

## Results

### Clinical characteristics

A total of 192 patients were approached to enroll 109 subjects including 93 who completed both study assessments and were analyzed (16 subjects died or refused the second assessment). For two subjects, there were <1,000 16S reads obtained from the swabs performed at ICU admission and these samples were excluded; the median sequence depth per sample for the remaining 184 samples exceeded 1,000,000.

Many subjects had evidence of hemodynamic instability at the time of ICU admission and most had anemia, leukocytosis, or renal insufficiency (S1 Table). Most patients were clinically improving during treatment in the ICU with a median decline of 12 points in the APACHE IV score (p<0.01) representing decreased severity of illness. However, over the 72 hours following ICU admission, patients were also significantly more likely to develop new fever, anemia, or hypoalbuminemia (S1 Table). During this initial 72 hour period, 77% of subjects received broad-spectrum antibiotics including 25% who received vancomycin (Table 1).

**Table 1:** Demographics and ICU interventions during the 72 hours following admission to the ICU.

**Demographics**	**N, %**
Sex	
Male	49 (53%)
Female	44 (47%)
Age	
<60 years old	32 (34%)
60–68	30 (32%)
>68 years old	31 (33%)
Admission type	
Medical	40 (43%)
Surgical	53 (57%)
**ICU Interventions**	**N, %**
Antibiotics	72 (77%)
PPIs	47 (44%)
Mechanical ventilation Some but <72 hrs ≥72 hours	23 (25%) 17 (18%)
Hemodialysis	6 (6%)
Enteral feeding	74 (80%)

ICU: intensive care unit

PPIs: proton pump inhibitors.

### Within-individual changes in the gut microbiome

**Fig 1:**
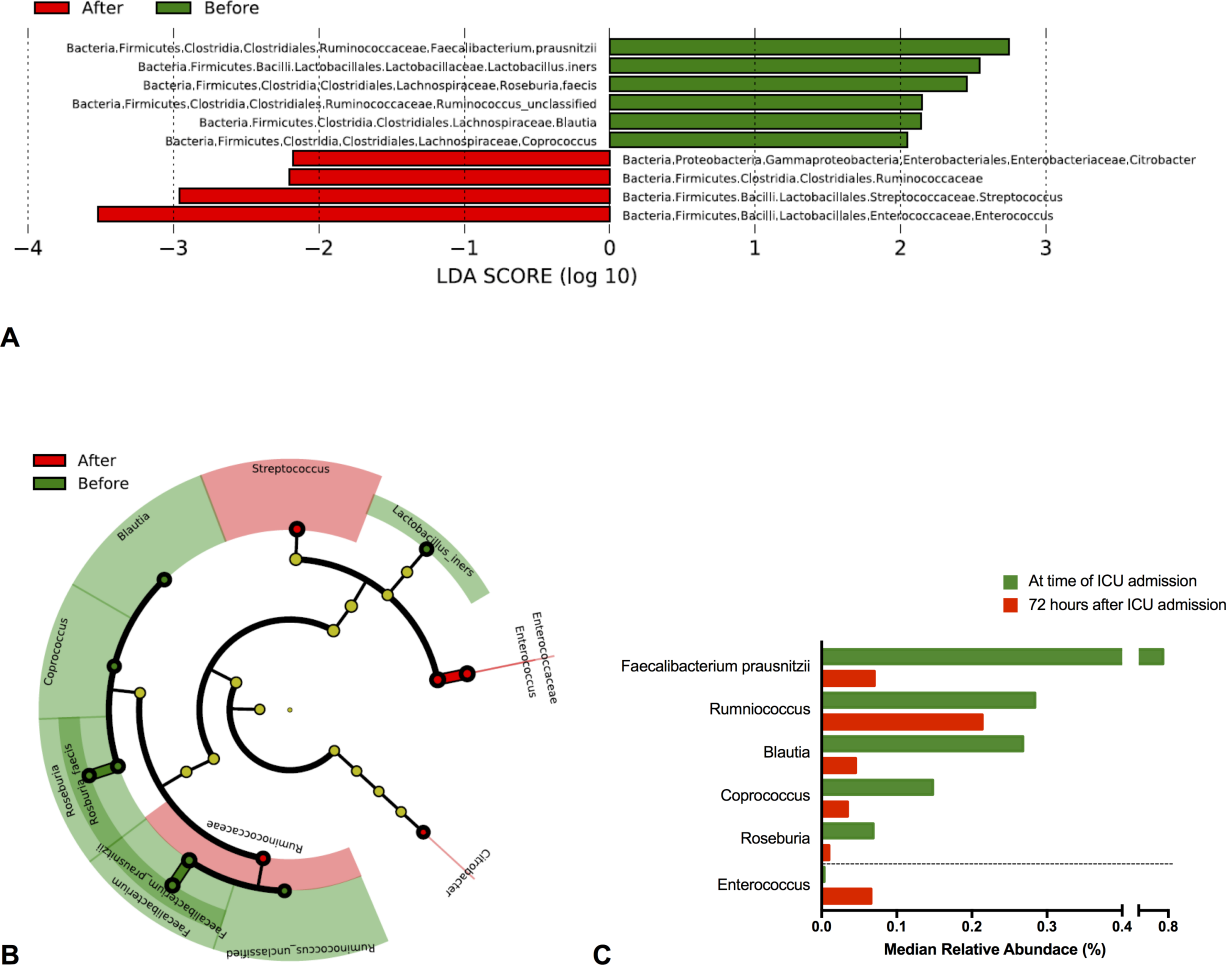
Changes within specific bacterial taxa, comparing ICU admission and 72 hours later. Using a linear discriminant analysis (LDA)-based algorithm, significant declines were identified in the short chain fatty acid-producing Clostridial Clusters IV (*Faecalibacterium prausnitzii*) and XIVa (*Blautia*, *Coprococcus*, *Roseburia*, and *Ruminococcus*) with an expansion in *Enterococcus*. Changes within specific bacterial taxa are shown as (A) LDA scores, which are proportional to the relative change within each of the bacterial taxa shown, (B) a cladogram, which shows the hierarchical relationship of these different taxa in relationship each other, and (C) relative abundance of these taxa. For (C) only taxa with a pooled minimum of 0.05% median relative abundance are depicted.

Over 72 hours, there was a modest within-individual decline in fecal microbial diversity that was not statistically significant (Shannon index, median change -3.5%, p = 0.06) and a significant decline in fecal microbial richness (operational taxonomic units, median change -7.1%, p<0.01). There were no major phylum-level shifts (S1 Fig). LEfSe was used to assess for within-individual changes in specific bacterial taxa over these initial 72 hours. There was a significant decline in the Clostridial Clusters IV (*Faecalibacterium prausnitzii*) and XIVa (*Blautia*, *Coprococcus*, *Roseburia*, and *Ruminococcus*) with an expansion in *Enterococcus* (Fig 1A–1C; complete LDA results in S2 Table). Among these Clostridia, the greatest decline was seen in *F*. *prausnitzii* which had an 8-fold median decline over 72 hours (Fig 1C).

### Altered Cluster IV/XIVa Clostridia

**Fig 2:**
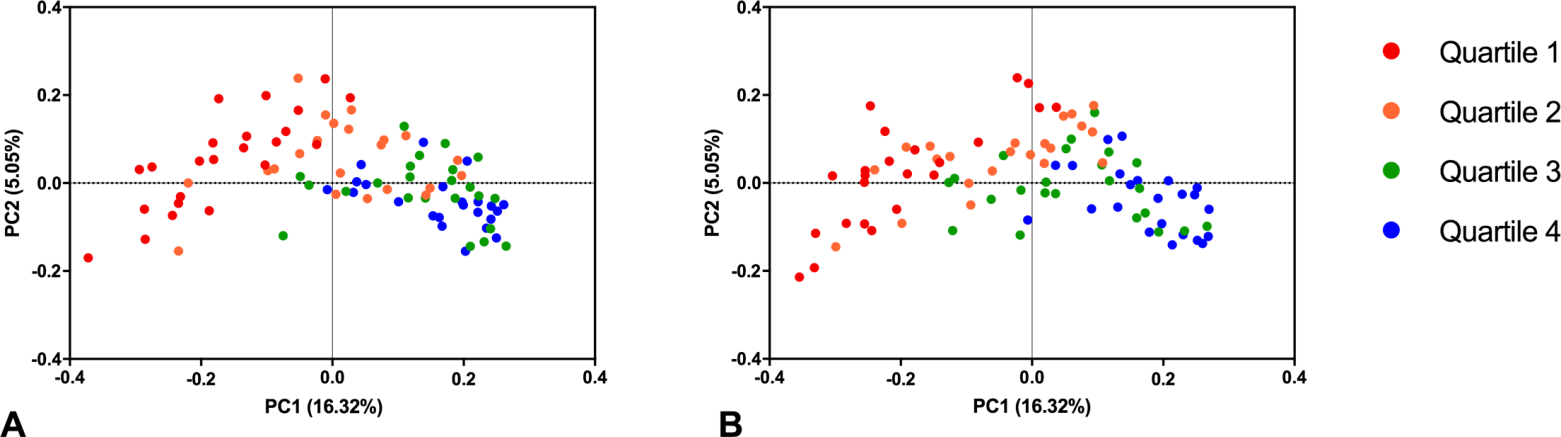
**Principal coordinates analysis (PCoA) of subjects at time of ICU admission (A) and 72 hours later (B), stratified by relative abundance Clostridial Clusters IV and XIVa**. There was distinct clustering of subjects based on levels of Clostridial Clusters IV and XIVa both at ICU admission and 72 hours later even though these Clostridia made up <1% overall relative abundance of the fecal microbiome. Taxa included in Cluster IV/XIVa: *F*. *prausnitzii*, *Blautia*, *Coprococcus*, *Roseburia*, and *Ruminococcus*. Subjects are color-coded by pooled levels of Cluster IV/XIVa Clostridia, from the lowest relative abundance (Quartile 1) to the highest relative abundance (Quartile 4).

**Fig 3:**
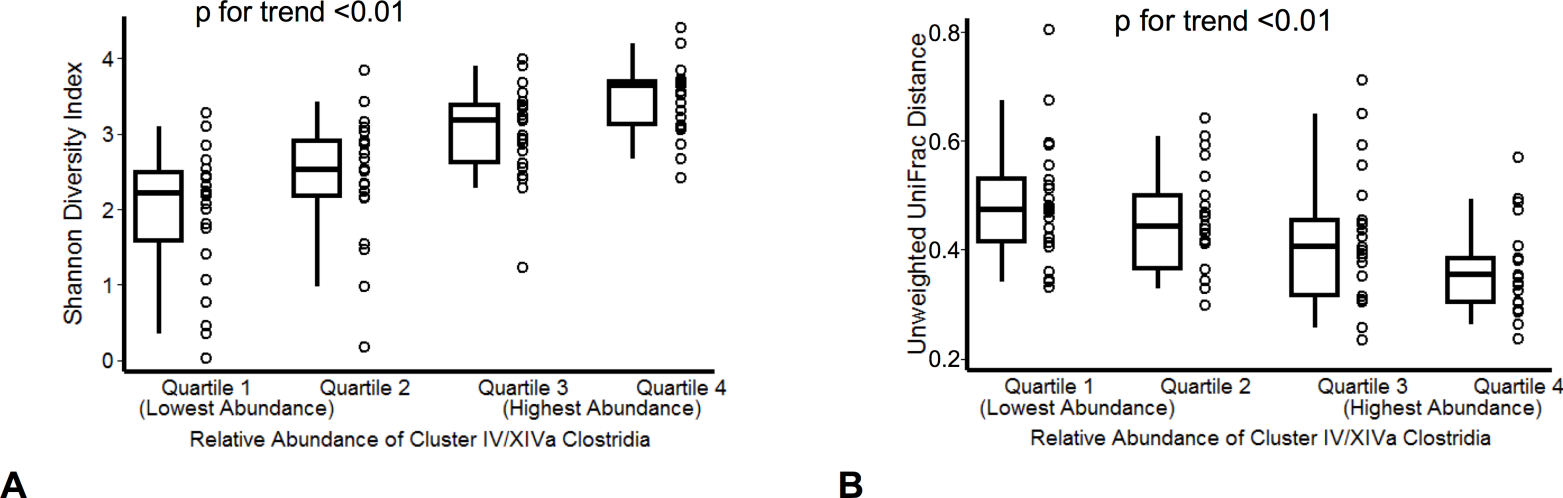
Relationship between Clostridial Clusters IV and XIVa and gut microbiome diversity and stability. Lower levels of Clostridial Clusters IV and XIVa at 72 hours were associated with decreased fecal biodiversity at 72 hours (A) and with decreased community stability over time (B). To test for a relationship between Cluster IV/XIVa Clostridia and fecal biodiversity, Shannon index was calculated for each sample and compared across levels of these taxa. To test for a relationship between Cluster IV/XIVa Clostridia and community stability, unweighted UniFrac distance was calculated for each subject comparing ICU admission to 72 hours later and compared across levels of these Clusters.

To understand the role of taxa found to be significantly altered in the ICU, the Cluster IV/XIVa Clostridia identified by LEfSe were pooled and organized into quartiles based on relative abundance, from lowest to highest. Median relative abundance was 2.6% at ICU admission versus 0.30% 72 hours later (sign-rank p = 0.01, see S2 Fig). On PCoA, there was clustering of subjects based on levels of Cluster IV/XIVa Clostridia both at the time of ICU admission (Fig 2A) and 72 hours later (Fig 2B) mirroring overall differences in microbial composition. Seventy-two hours after admission, lower levels of Cluster IV/XIVa Clostridia were associated with decreased diversity (Fig 3A).

Additionally, low levels of Cluster IV/XIVa Clostridia at 72 hours were associated with decreased within-individual community stability between ICU admission and 72 hours later as measured by unweighted UniFrac distance (Fig 3B).

### Relationship between *Enterococcus* and Cluster IV/XIVa Clostridia

**Fig 4:**
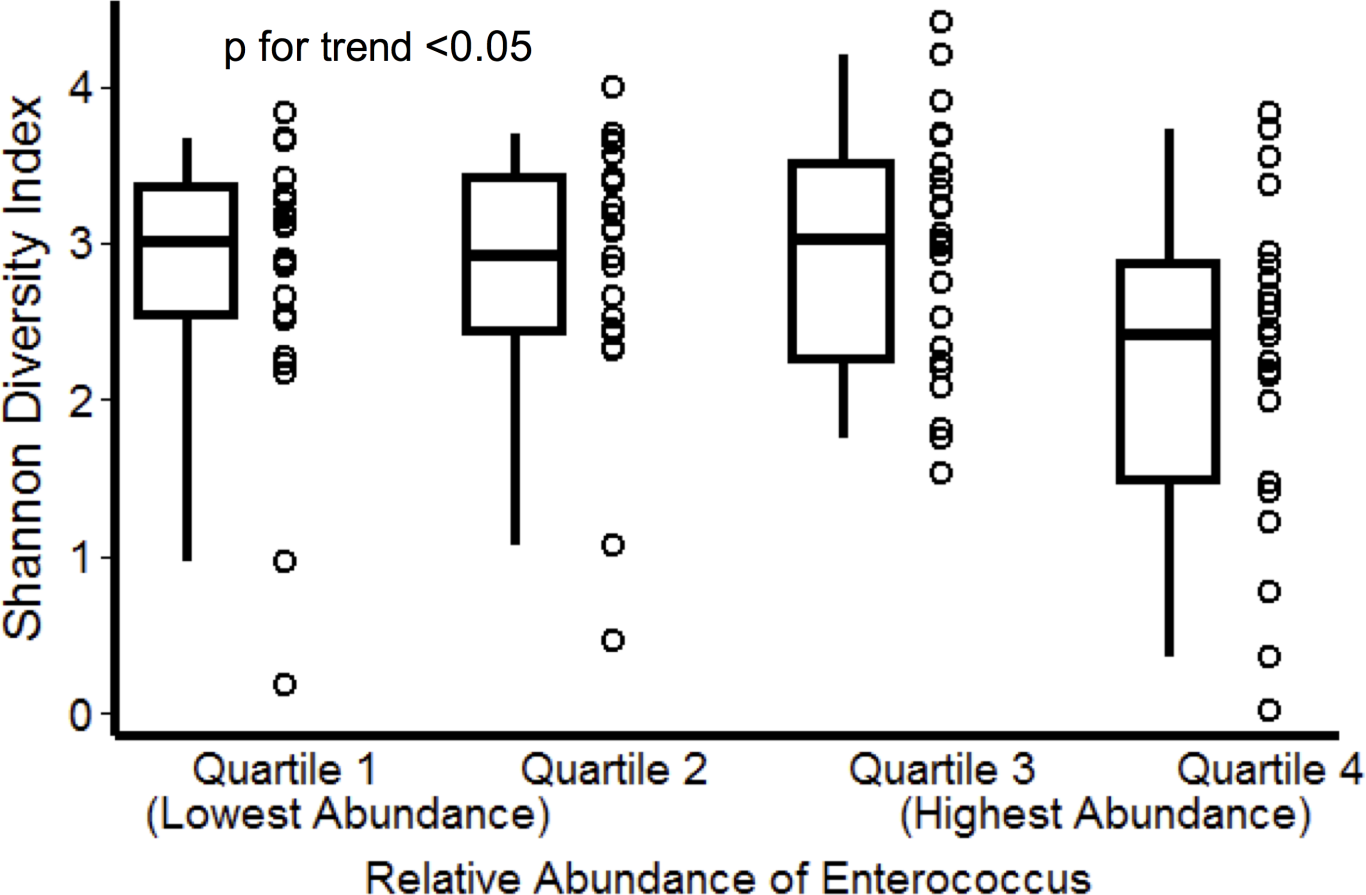
Fecal microbial diversity and *Enterococcus* after 72 hours in the intensive care unit (ICU). Higher levels of *Enterococcus* were associated with decreased fecal biodiversity (Shannon Index).

*Enterococcus*, which was found to be significantly increased on LEfSe analysis (Fig 1A–1C), was organized into quartiles. Median relative abundance of *Enterococcus* was 0.0038% at ICU admission and 0.067% 72 hours later (sign-rank p<0.01, see S2 Fig). Higher levels of *Enterococcus* at 72 hours were associated with decreased diversity (Fig 4). There was evidence of a reciprocal relationship where high levels of *Enterococcus* were only observed with low levels of Cluster IV/XIVa Clostridia (S3A Fig) and PCoA showed distinct clusters based on high levels of Cluster IV/XIVa Clostridia or high levels of *Enterococcus*, without overlap between clusters (S3B Fig). 16S rRNA gene sequencing results do not distinguish between vancomycin-sensitive versus vancomycin-resistant *Enterococcus* (VRE), so VRE culture was performed to assess the potential clinical importance of *Enterococcus* from 16S rRNA gene sequencing data. The relative abundance of *Enterococcus* based on 16S rRNA gene sequencing was highly associated with VRE culture positivity (p<0.01, S4 Fig). There were 23 patients (25%) who received vancomycin during the initial 72 hours of ICU treatment and among these patients, VRE rates in culture after 72 hours were 22% compared to 7% for subjects who did not receive vancomycin (Fisher’s p = 0.11) *Enterococcus* relative abundance after 72 hours was over 6-fold higher in subjects who received vancomycin compared to those who did not (rank-sum p = 0.05).

### Multivariable models

Ordinal logistic regression was used to model the relationship between clinical characteristics and taxa found to be altered on LEfSe, with patients classified across 4 ranked quartiles of relative abundance of Cluster IV/XIVa Clostridia or *Enterococcus* from lowest to highest. Receipt of antibiotics and anemia were significantly associated with decreased Cluster IV/XIVa Clostridia (Table 2). Assessing for dynamic changes, the development of new fever was also associated with decreased Cluster IV/XIVa Clostridia after 72 hours and was included in the final model. When diversity (Shannon index) was added to the final model as a linear variable, the relationship between receipt of antibiotics and Cluster IV/XIVa Clostridia lost significance (OR 2.15, 95% CI 0.77–6.04). When patients with documented gastrointestinal bleeding were excluded, there was no change in the relationship between anemia and levels of Cluster IV/XIVa Clostridia (OR 3.93, 95% CI 1.37–11.3). None of the model estimates were altered by adjusting for acute severity of illness. Clinical characteristics were examined in relationship to the relative abundance of *Enterococcus* after 72 hours and only receipt of antibiotics was significantly associated with increased *Enterococcus* (Table 3).

**Table 2:** Relationship between clinical variables and decreased Clostridial Clusters IV and XIVa after 72 hours in the ICU.

Variable	Unadjusted OR (95% CI) *	Multivariable OR (95% CI) *
Demographics		
Female sex	1.17 (0.57–2.43)	—
Age		
<60 years old	Ref	Ref
60–68 years old	0.60 (0.24–1.46)	—
>68 years old	0.82 (0.33–2.04)	—
Surgical admission**	1.29 (0.62–2.72)	—
Clinical characteristics after 72 hours		
Fever (temp >38°C)		
Newly developing	2.84 (0.60–13.6)	5.07 (1.02–25.2)
After 72 hours	2.61 (0.65–10.5)	—
Hematocrit <33.8%	5.36 (2.11–13.6)	5.08 (1.77–14.6)
WBC ≥12,000 x 10^9^/L	0.96 (0.46–2.01)	—
Albumin <3.5 g/dL	2.18 (1.00–4.76)	1.07 (0.44–2.60)
APACHE IV score	1.00 (0.99–1.02)	—
Treatment-related characteristics over 72 hours		
Antibiotics (any)	3.86 (1.47–10.1)	2.90 (1.06–7.91)
PPIs (any)	1.92 (0.91–4.02)	—
Ventilation ≥72 Hours	1.28 (0.51–3.26)	—
Hemodialysis	1.53 (0.41–5.76)	—
Enteral feeding	0.89 (0.37–2.15)	—

ICU: intensive care unit; OR: odds ratio; CI: confidence interval; WBC: white blood cell count; APACHE: acute physiology and chronic health evaluation. Shown are patient characteristics that significantly changed during the 72 hours after ICU admission and all treatment-related variables. Boldfaced variables were significant at p<0.05 in the final model.

*Ordinal logistic regression: ORs represent odds of moving from a higher into a lower quartile of relative abundance of Clostridial Clusters IV and XIVa.

**Comparator: medical admission.

**Table 3:** Relationship between clinical variables and increased *Enterococcus* after 72 hours in the ICU.

Variable	Unadjusted OR* (95% CI)	Multivariable OR* (95% CI)
Demographics		
Female sex	1.05 (0.51–2.18)	—
Age		
<60 years old	Ref	Ref
60–68 years old	0.78 (0.32–1.88)	—
>68 years old	0.88 (0.36–2.16)	—
Surgical admission**	0.50 (0.23–1.06)	—
Clinical characteristics after 72 hours		
Fever (temp >38°C)		
Newly developing	0.48 (0.12–1.98)	—
After 72 hours	0.51 (0.10–2.54)	—
Hematocrit <33.8%	1.65 (0.72–3.81)	—
WBC ≥12,000 x 10^9^/L	1.30 (0.61–2.75)	—
Albumin <3.5 g/dL	1.24 (0.59–2.61)	—
APACHE IV score	1.02 (1.00–1.03)	1.00 (0.98–1.02)
Treatment-related characteristics over 72 hours		
Antibiotics (any)	3.19 (1.33–7.67)	2.66 (1.08–6.57)
PPIs (any)	0.69 (0.33–1.44)	—
Ventilation ≥72 Hours	3.51 (1.26–9.79)	1.49 (0.32–6.94)
Hemodialysis	1.03 (0.21–4.99)	—
Enteral feeding	0.31 (0.12–0.79)	0.40 (0.15–1.11)

ICU: intensive care unit; OR: odds ratio; CI: confidence interval; WBC: white blood cell count; APACHE: acute physiology and chronic health evaluation. Shown are patient characteristics that significantly changed during the 72 hours after ICU admission and all treatment-related variables. Boldfaced variables were significant at p<0.05 in the final model.

*Ordinal logistic regression: ORs represent odds of moving from a higher into a lower quartile of relative abundance of *Enterococcus*.

**Comparator: medical admission.

In addition to the above approach using quartiles, linear regression analysis was performed with the relative abundance for *Enterococcus* and Clostridial Clusters IV/XIVa classified as continuous, log-transformed linear measures. These results were qualitatively similar although effect size estimates increased. After adjusting for other clinical variables, antibiotics were again the most important risk factor associated with increased relative abundance of *Enterococcus* (OR 5.81, 95% CI 1.17–28.7) and with decreased relative abundance of Clostridial Clusters IV/XIVa (OR 8.01, 95% CI 1.71–36.6).

## Discussion

In this prospective cohort study, initial treatment in the ICU was accompanied by a rapid loss of short chain fatty acid (SCFA)-producing bacteria within Clostridial Clusters IV (*Faecalibacterium prausnitzii*) and XIVa (*Blautia*, *Coprococcus*, *Roseburia*, and *Ruminococcus*) [30]. Loss of these SCFA-producing Clostridia was associated with low overall bacterial biodiversity, with evidence of community instability and with an expansion in *Enterococcus* (including VRE). These data suggest that loss of Cluster IV/XIVa Clostridia in the ICU may be associated with deleterious changes within the structure of the fecal microbiome and these bacteria may be good candidates for further study in the critical care setting, especially studies seeking to determine if these candidate species can predict adverse outcomes in the ICU.

SCFA-producing Clostridial Clusters were identified based on an untargeted analysis demonstrating within-individual loss of these taxa with a concurrent rise in *Enterococcus* (including VRE). These findings are consistent with prior studies which demonstrate the importance of these organisms for maintenance of gut homeostasis [13] and extend this finding into the ICU setting. In a recent prior study comparing the fecal microbiome of 10 ICU patients to healthy subjects, there was a decreased abundance of Clostridia clusters IV/XIa and increased abundance of *Enterococcus* [31]. In another cohort of 34 ICU patients, there was a lower abundance of *Faecalibacterium*, *Ruminococus and Blautia*, all taxa in Clostridia clusters IV/XIV, compared to healthy subjects [32]. The current study extends these findings to a larger ICU cohort, and assesses dynamic changes within these taxa. Recent data also support the idea that SCFA producers are important in colonization resistance against VRE. Specifically, butyrate producing bacteria may restrict the outgrowth of potential pathogens through intracellular butyrate sensor peroxisome proliferator-activated receptor γ (PPAR-γ) and by limiting luminal oxygen [33]. Cluster XIVa Clostridia (*Blautia producta* and *Clostridium Bolteae*) prevents VRE colonization in antibiotic-treated mice and inhibition of VRE colonization seems to be the result of a direct interaction between *B*. *producta* and VRE [34]. In BMT patients, low fecal microbial diversity at the time of BMT predicted VRE bacteremia and mortality [8, 9, 35]. In the ICU, prolonged stays have been associated with a progressive loss of Clostridia with replacement by *Enterococcus* [7] and low fecal SCFA levels have been correlated with domination by *Enterococcus* and with worse clinical outcomes [14, 36]. Our study highlights the potential importance of SCFA producers in preventing VRE colonization in an ICU-based population.

In addition to VRE, we found inverse relationships between Cluster IV/XIVa Clostridia and other potential pathogens including *Staphylococcus* and *Enterobacteriaceae*. Prior studies support the hypothesis that SCFA producers have a role in gut microbiome homeostasis that extends beyond colonization resistance against VRE [37]. SCFAs are produced from dietary fiber and have been linked to decreased colonic inflammation [38], an enlarged pool of regulatory T cells [39], and beneficial effects on energy metabolism [40]. In animals, fiber deprivation led to thinning of the colonic mucus layer and increased pathogen susceptibility [41]. In BMT patients, taxa within Cluster IV/XIVa Clostridia were associated with decreased risk for *Clostridium difficile* infection [42]. In ICU patients sampled within 48 hours of admission and at ICU discharge, loss of *Faecalibacterium* was associated with loss of diversity and outgrowth of Enterobacteriaceae [15, 43]. In our data, Cluster IV/XIVa Clostridia comprised a small proportion of the total gut microbiome by abundance yet explained a large degree of inter-individual variability on clustering analyses and were highly correlated with diversity and community stability, typical markers for a healthy gut microbiome across populations [44, 45]. These findings imply that these bacteria may serve a critical functional role in the distal gut. Yet fiber, the primary fuel substrate for the Clostridia identified here, is often missing from the enteral feeds used in the ICU [46].

In this data, receipt of antibiotics was associated with low levels of Cluster IV/XIVa Clostridia and with high levels of *Enterococcus*. Subjects who received antibiotics had increased levels of *Enterococcus* on sequencing, and subjects who received vancomycin had a 15% absolute increase in rates of VRE colonization based on culture. Approximately 75% of ICU patients receive antibiotics for known or suspected infections [47, 48] and, often broad-spectrum antibiotics are given before the results of cultures or other testing become available. Our data support the idea that antibiotics induce broad structural changes within the gut microbiome and is consistent with the observation that antibiotics promote colonization with antibiotic-resistant bacteria [49]. However, allocation of antibiotics was not random, and it is also possible that unidentified patient characteristics determined both use of antibiotics and the microbiome changes that we observed.

There is substantial interest in testing pre- or probiotics to prevent infections in the ICU. In murine models, defined mixtures of bacteria including bacteria within the Clostridial Clusters IV/XIVa provided colonization resistance to VRE after antibiotic challenge [50] and promoted VRE intestinal clearance [34]. In germ-free mice, a consortium of Clostridia including Clusters IV/XIVa conferred colonization resistance to the enteric pathogens *S*. *Typhimurium* and *C*. *rodentium* [51]. In a murine sepsis model, there was a survival benefit with fiber supplementation, mediated in part by a rise in Cluster XIVa Clostridia [52]. To date, clinical trials testing probiotics have delivered heterogeneous organisms (e.g., *Lactobacillus* or *Bifidobacterium sp*.) and have had mixed results or in some cases have been associated with harm [53–55]. Future studies may wish to address whether SCFA-producing Clostridia, or related prebiotics, can be successfully delivered and retained within the colonic microbiome.

Our study has several strengths. There was a rigorous protocol for sample collection, with subjects sampled immediately at the time of ICU admission and again 72 hours later. This serial sampling permitted evaluation for dynamic within-individual changes over the initial ICU treatment period. A broad swath of clinical data was ascertained both at ICU admission and 72 hours later, which allowed us to assess relationships after adjusting for underlying patient factors. Standard bacterial culture for VRE was used to confirm sequencing results related to *Enterococcus*, the main pathogen showing outgrowth in these subjects.

There are also limitations. Because we sought to acquire samples immediately at the time of ICU admission, it was not possible to gather whole stools and therefore not possible to directly assay levels of SCFAs. Despite this early acquisition of samples, potentially protective bacterial taxa may be lost prior to ICU admission and potentially pathogenic bacteria may be gained [56]. Our approach focused on ICU admission and 72 hours later because by 72 hours the clinical outcomes of most patients are determined [57]. This study was conducted in multiple ICUs within a single tertiary care center and the prevalence of specific organisms and antibiotic susceptibilities is likely to differ at other institutions. Finally, our conclusions should be tempered by recognizing that there is a dense network of interdependency between gut commensals [58]. No single bacterial species, and probably no single functionally related group of bacteria, is likely to be completely responsible for colonization resistance.

In summary, we found that Clostridia within the Clusters IV/XIVa were rapidly lost during the initial treatment period in the ICU. Low levels of Cluster IV/XIVa Clostridia were associated with receipt of antibiotics, but no other potentially modifiable ICU interventions. Low levels of Cluster IV/XIVa Clostridia were also associated with low fecal microbial diversity, microbiome community instability, and the outgrowth of VRE. SCFA-producing Clostridia may serve a role in the maintenance of normal gastrointestinal colonization resistance in the ICU.

## Supporting information

S1 Fig Changes within the fecal microbiome following intensive care unit (ICU) admission. (PDF)

S2 Fig Histogram showing within-individual change in *Enterococcus* (A) and Clostridial Clusters IV and XIVa (B) during the 72 hours after ICU admission.
(PDF)

S3 Fig Relationship between Enterococcus and Clostridial Clusters IV and XIVa after 72 hours in the ICU. (PDF)

S4 Fig Proportion of *Enterococcus* in 16S rRNA gene sequencing results, according to vancomycin-resistant *Enterococcus* (VRE) culture results. (PDF)

S1 File Supplementary materials and methods. (PDF)

S1 Table Patient characteristics at the time of admission to the intensive care unit and 72 hours later. (PDF)

S2 Table LDA scores and p-values for discriminative taxa identified using LEfSe. (PDF)
