# Supplementary material for: Rapid gastrointestinal loss of Clostridial Clusters IV and XIVa in the ICU associates with an expansion of gut pathogens
Source: PLoS One. 2018 Aug 1;13(8):e0200322. doi: 10.1371/journal.pone.0200322 (PMC6070193; doi:10.1371/journal.pone.0200322)
Supplement: S1 Fig — (PDF) [file pone.0200322.s001.pdf]

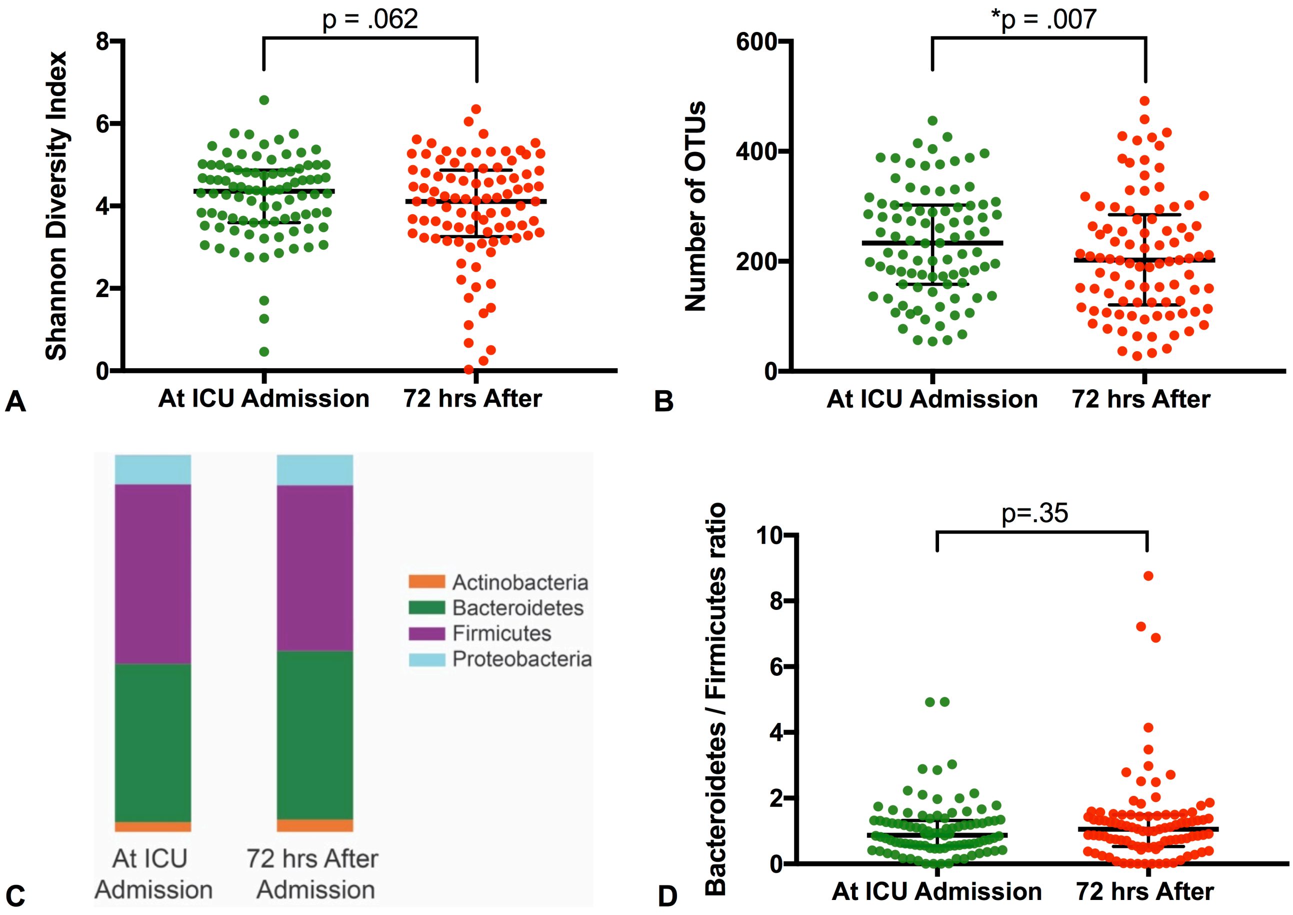

S1 Fig. Changes within the fecal microbiome following intensive care unit (ICU) admission. Over the 72 hours following admission to the ICU, there was a modest decline in fecal microbial diversity (A) and in the number of operational taxonomic units (OTUs) (B). All samples rarefied to depth of 50,000 for these analyses. There were no major phylum level shifts (p>0.1) for each phylum (C) or in testing for changes in the Bacteroidetes to Firmicutes ratio (D).
