# Supplementary material for: Rapid gastrointestinal loss of Clostridial Clusters IV and XIVa in the ICU associates with an expansion of gut pathogens
Source: PLoS One. 2018 Aug 1;13(8):e0200322. doi: 10.1371/journal.pone.0200322 (PMC6070193; doi:10.1371/journal.pone.0200322)
Supplement: S2 Fig — Histogram showing within-individual change in Enterococcus (A) and Clostridial Clusters IV and XIVa (B) during the 72 hours after ICU admission. (PDF) [file pone.0200322.s002.pdf]

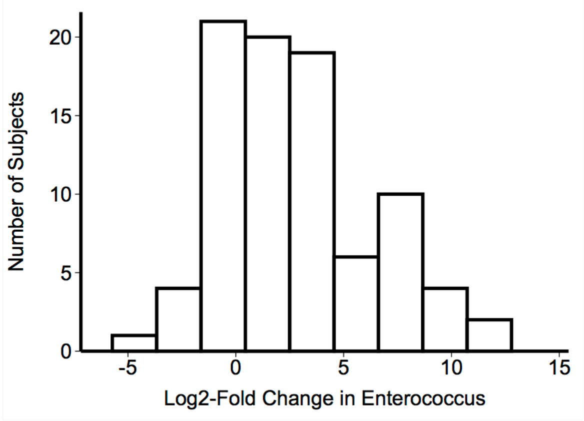

A

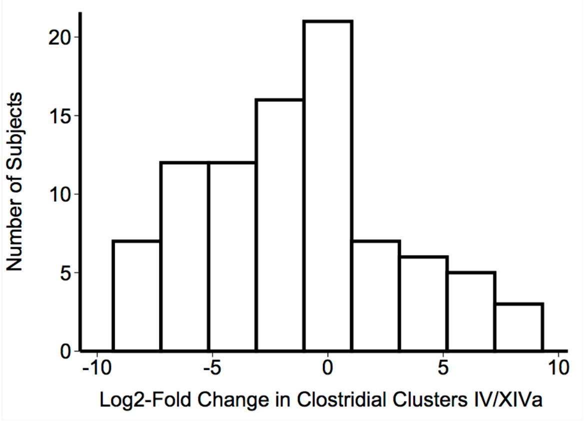

B

S2 Fig. Histogram showing within-individual change in *Enterococcus* (A) and Clostridial Clusters IV and XIVa (B) during the 72 hours after ICU admission. (A) For all individuals, the within-individual change in *Enterococcus* was calculated between ICU admission and sample collection 72 hours later. The histogram is plotted on a log2 scale, so not shown are three subjects who had undetectable *Enterococcus* at admission and an additional 3 subjects who had undetectable *Enterococcus* after 72 hours. (B) For all individuals, the within-individual change in Clostridial Clusters IV/XIVa was calculated between ICU admission and sample collection 72 hours later. The histogram is plotted on a log2 scale, so not shown is one subject who had undetectable Clostridial Clusters IV/XIVa at admission and an additional 4 subjects who had undetectable IV/XIVa after 72 hours.
