# Supplementary material for: Rapid gastrointestinal loss of Clostridial Clusters IV and XIVa in the ICU associates with an expansion of gut pathogens
Source: PLoS One. 2018 Aug 1;13(8):e0200322. doi: 10.1371/journal.pone.0200322 (PMC6070193; doi:10.1371/journal.pone.0200322)
Supplement: S3 Fig — (PDF) [file pone.0200322.s003.pdf]

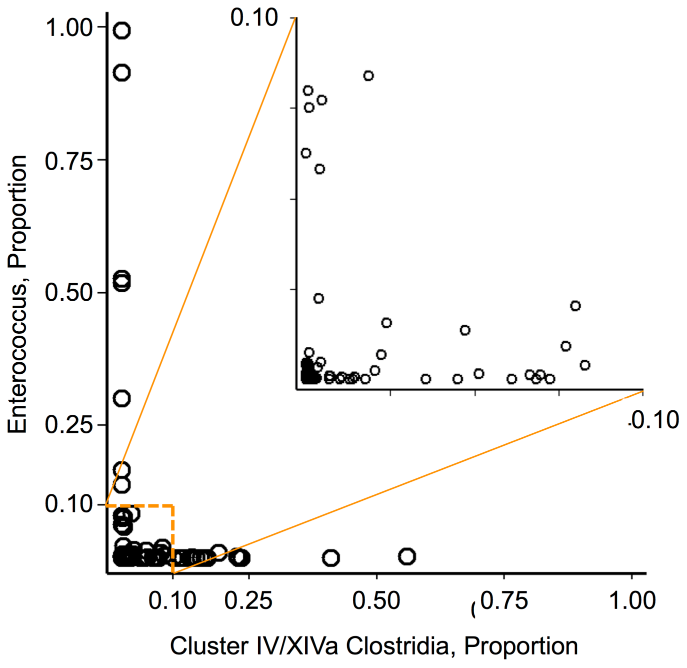

A

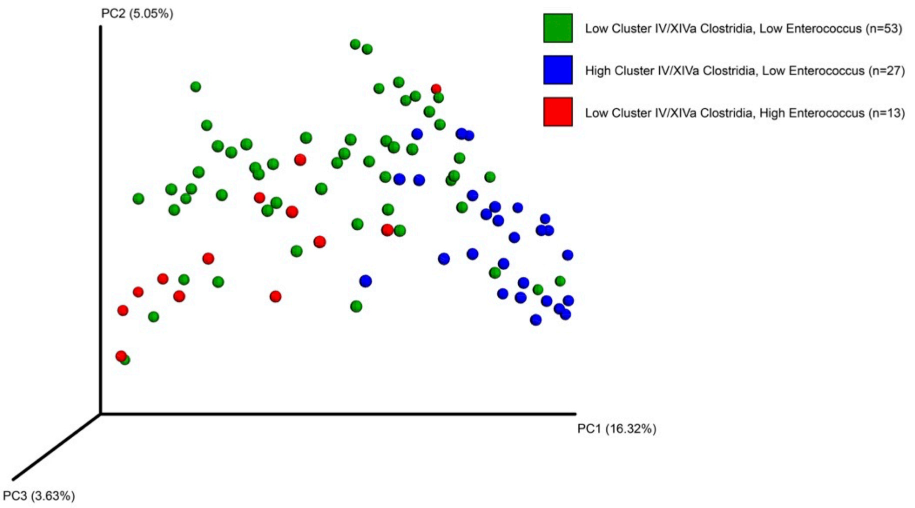

B

S3 Fig. Relationship between Enterococcus and Clostridial Clusters IV and XIVa after 72 hours in the ICU. There was evidence of a reciprocal relationship between *Enterococcus* and Clostridial Clusters IV and XIVa where high levels of *Enterococcus* were only observed in the setting of low levels of these Clostridia. (A) Relative abundance of *Enterococcus* as a function of Cluster IV/XIVa Clostridia. Total relative abundance cannot exceed 1.0, so domination by Cluster IV/XIVa Clostridia excludes the possibility of high levels of *Enterococcus*. However, the same effect was observed in patients where the relative abundance of both groups of taxa was <0.1 (inset). (B) Principal coordinates analysis of unweighted UniFrac distances. Based on the observed data, we colored samples based on high Cluster IV/XIVa Clostridia samples (>0.025 relative abundance) and low (≤0.025), and on high in *Enterococcus* (>0.025) and low (≤0.025). There were no samples with both high Cluster IV/XIVa Clostridia and high *Enterococcus*. There was no overlap between the samples high in Cluster IV/XIVa Clostridia (blue) and those high in *Enterococcus* (red).
