# Supplementary material for: Rapid gastrointestinal loss of Clostridial Clusters IV and XIVa in the ICU associates with an expansion of gut pathogens
Source: PLoS One. 2018 Aug 1;13(8):e0200322. doi: 10.1371/journal.pone.0200322 (PMC6070193; doi:10.1371/journal.pone.0200322)
Supplement: S1 File — (PDF) [file pone.0200322.s005.pdf]

Supplementary materials and methods

Primer sequences and PCR protocol

Primer	Sequence
515_f1	GAGTTCAGACGTGTGCTCTTCCGATCT GTGCCAGCMGCCGCGGTAA
806_r1_N3	CCTACACGACGCTCTTCCGATCT NNN GGACTACHVGGGTWTCTAAT
806_r1_N4	CCTACACGACGCTCTTCCGATCT NNNN GGACTACHVGGGTWTCTAAT
806_r1_N5	CCTACACGACGCTCTTCCGATCT NNNNN GGACTACHVGGGTWTCTAAT
806_r1_N6	CCTACACGACGCTCTTCCGATCT NNNNNN GGACTACHVGGGTWTCTAAT
P5_r2	AATGATACGGCGACCACCGAGATCT ACAC TCTTTC CCTACACGACGCTCTTCCGATCT

PCR Protocol

Low-cycle PCR was performed using KAPA SYBR FAST qPCR (Kapa Biosystems, Wilmington, MA) per the manufacturer's instruction. Nested PCR cycles were performed using the following protocol on a Bio-Rad CFX96 Touch real-time PCR instrument (Bio-Rad, Hercules, CA).

PCR 1

Starting template: 20 ng DNA.
Primer sets: 515_f1 and 806_r1_N3-6.

Step 1 at 95 ºC for 3 min;
Step 2 at 95 ºC for 10 sec;
Step 3 at 60 ºC for 30 sec;
Repeat steps 2 x 3-30 cycles;
Step 5 at 68 ºC for 5 min;
Step 6 at 4 ºC on hold.

PCR 2

Starting template: 1 µl of PCR product.
Primer sets: P7_bc01-48 and P5_r2 with P5/P7 adaptors.

Step 1 at 95 ºC for 3 min;
Step 2 at 95 ºC for 10 sec;
Step 3 at 60 ºC for 30 sec;
Repeat steps 2 through 3 x 10 cycles;
Step 5 at 68 ºC for 5 min;
Step 6 at 4 ºC on hold.

Supplementary References

1. Caporaso JG, Lauber CL, Walters WA, Berg-Lyons D, Lozupone CA, Turnbaugh PJ, et al. Global patterns of 16S rRNA diversity at a depth of millions of sequences per sample. Proc Natl Acad Sci U S A. 2011;108 Suppl 1:4516-22. Epub 2010/06/11. doi: 10.1073/pnas.1000080107. PubMed PMID: 20534432; PubMed Central PMCID: PMCPMC3063599.

2. Human Microbiome Project C. Structure, function and diversity of the healthy human microbiome. Nature. 2012;486(7402):207-14. Epub 2012/06/16. doi: 10.1038/nature11234. PubMed PMID: 22699609; PubMed Central PMCID: PMCPMC3564958.
