# Supplementary material for: Rapid gastrointestinal loss of Clostridial Clusters IV and XIVa in the ICU associates with an expansion of gut pathogens
Source: PLoS One. 2018 Aug 1;13(8):e0200322. doi: 10.1371/journal.pone.0200322 (PMC6070193; doi:10.1371/journal.pone.0200322)
Supplement: S1 Table — (PDF) [file pone.0200322.s006.pdf]

S1 Table. Patient characteristics at the time of admission to the intensive care unit and 72 hours later.

Characteristics	ICU Admission (n, %)	After 72 Hours (n, %)	P-value for Change
Clinical characteristics			
Fever (temp >38ºC)			
Yes	17 (18%)	6 (7%)	0.02
No	76 (82%)	87 (94%)	
Tachycardia (HR >90 bpm)			
Yes	37 (40%)	33 (36%)	0.48
No	56 (60%)	60 (65%)	
Oliguria (UOP <800 cc/24h)			
Yes	14 (15%)	13 (14%)	0.82
No	79 (85%)	80 (86%)	
Laboratory values			
Hematocrit			
<33.8%	49 (53%)	69 (74%)	<0.01
≥33.8%	44 (47%)	24 (26%)	
White blood cell count			
≥12,000 cells x 10^9/L	42 (45%)	34 (37%)	0.10
<12,000 cells x 10^9/L	51 (55%)	59 (63%)	
Creatinine			
>1.3 mg/dL	23 (25%)	28 (30%)	0.13
≤1.3 mg/dL	70 (75%)	65 (70%)	
Sodium			
>145 or <137 mEq/L	25 (27%)	34 (37%)	0.11
137-145 mEq/L	68 (73%)	59 (63%)	
Total bilirubin			
>1.3 mg/dL	19 (20%)	19 (20%)	1.00
≤1.3 mg/dL	74 (80%)	74 (80%)	
Albumin			
<3.5 g/dL	43 (46%)	58 (62%)	<0.01
≥3.5 g/dL	50 (54%)	35 (38%)	
APACHE IV* score	54 (38-88)	42 (33-59)	<0.01

*Calculated without age.
