# Supplementary material for: Rapid gastrointestinal loss of Clostridial Clusters IV and XIVa in the ICU associates with an expansion of gut pathogens
Source: PLoS One. 2018 Aug 1;13(8):e0200322. doi: 10.1371/journal.pone.0200322 (PMC6070193; doi:10.1371/journal.pone.0200322)
Supplement: S2 Table — (PDF) [file pone.0200322.s007.pdf]

S2 Table. LDA scores and p-values for discriminative taxa identified using LEfSe.

Discriminative taxa	Group Enriched	Log2-Fold Change (Median)	LDA score	P-value
Bacteria.Firmicutes.Bacilli.Lactobacillales.Enterococcaceae.Enterococcus	After 72 hours	+2.2	3.522	0.000
Bacteria.Firmicutes.Bacilli.Lactobacillales.Streptococcaceae.Streptococcus	After 72 hours	+.89	2.959	0.048
Bacteria.Firmicutes.Clostridia.Clostridiales.Lachnospiraceae.Roseburia	At ICU admission	-1.6	2.632	0.015
Bacteria.Firmicutes.Clostridia.Clostridiales.Ruminococcaceae.Faecalibacterium.prausnitzii	At ICU admission	-1.2	2.743	0.035
Bacteria.Firmicutes.Clostridia.Clostridiales.Ruminococcaceae	At ICU admission	-1.8	2.204	0.040
Bacteria.Proteobacteria.Gammaproteobacteria.Enterobacteriales.Enterobacteriaceae.Citrobacter	After 72 hours	+2.2	2.178	0.037
Bacteria.Firmicutes.Clostridia.Clostridiales.Lachnospiraceae.Blautia	At ICU admission	-1.2	2.143	0.031
Bacteria.Firmicutes.Clostridia.Clostridiales.Lachnospiraceae.Coprococcus	At ICU admission	-.65	2.047	0.013

Taxa have been ordered by LDA score from highest to lowest. LDA: least discriminant analysis; LEfSe: LDA effect size algorithm.
