# Supplementary material for: Rapid gastrointestinal loss of Clostridial Clusters IV and XIVa in the ICU associates with an expansion of gut pathogens
Source: PLoS One. 2018 Aug 1;13(8):e0200322. doi: 10.1371/journal.pone.0200322 (PMC6070193; doi:10.1371/journal.pone.0200322)
Supplement: S4 Fig — (PDF) [file pone.0200322.s004.pdf]

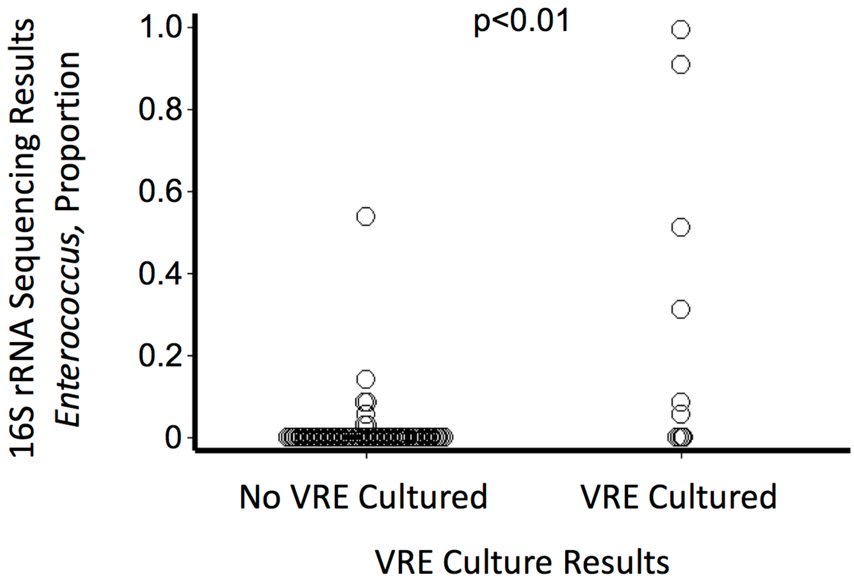

S4 Fig. Proportion of *Enterococcus* in 16S rRNA gene sequencing results, according to vancomycin-resistant *Enterococcus* (VRE) culture results. The relative abundance of *Enterococcus* by 16S rRNA sequencing results was highly associated with VRE culture positivity from rectal swabs taken at the same time.
